# Screen-Printed Graphite Electrodes as Low-Cost Devices for Oxygen Gas Detection in Room-Temperature Ionic Liquids

**DOI:** 10.3390/s17122734

**Published:** 2017-11-26

**Authors:** Junqiao Lee, Ghulam Hussain, Craig E. Banks, Debbie S. Silvester

**Affiliations:** 1 Curtin Institute for Functional Molecules and Interfaces & Department of Chemistry, Curtin University, GPO Box U1987, Perth, WA 6845, Australia; juniaiko@gmail.com (J.L.); ghulam.hussain1985@gmail.com (G.H.); 2 Faculty of Science and Engineering, Manchester Metropolitan University, Chester Street, Manchester M1 5GD, UK; C.Banks@mmu.ac.uk

**Keywords:** screen-printed electrode, graphite, room temperature ionic liquids, gas sensing, oxygen reduction, ink formulation

## Abstract

Screen-printed graphite electrodes (SPGEs) have been used for the first time as platforms to detect oxygen gas in room-temperature ionic liquids (RTILs). Up until now, carbon-based SPEs have shown inferior behaviour compared to platinum and gold SPEs for gas sensing with RTIL solvents. The electrochemical reduction of oxygen (O_2_) in a range of RTILs has therefore been explored on home-made SPGEs, and is compared to the behaviour on commercially-available carbon SPEs (C-SPEs). Six common RTILs are initially employed for O_2_ detection using cyclic voltammetry (CV), and two RTILs ([C_2_mim][NTf_2_] and [C_4_mim][PF_6_]) chosen for further detailed analytical studies. Long-term chronoamperometry (LTCA) was also performed to test the ability of the sensor surface for real-time gas monitoring. Both CV and LTCA gave linear calibration graphs—for CV in the 10–100% vol. range, and for LTCA in the 0.1–20% vol. range—on the SPGE. The responses on the SPGE were far superior to the commercial C-SPEs; more instability in the electrochemical responses were observed on the C-SPEs, together with some breaking-up or dissolution of the electrode surface materials. This study highlights that not all screen-printed ink formulations are compatible with RTIL solvents for longer-term electrochemical experiments, and that the choice of RTIL is also important. Overall, the low-cost SPGEs appear to be promising platforms for the detection of O_2_, particularly in [C_4_mim][PF_6_].

## 1. Introduction

The detection of oxygen gas at higher (percent) concentrations is of wide significance in a range of industries, including mining [1], medical [2], food quality [3], environmental monitoring [4], and space exploration [5]. From a personal safety perspective, concentrations of oxygen below 18% vol. are potentially dangerous due to an increasing risk of asphyxiation and death, and concentrations in excess of 23% present a risk of fire and explosion [6]. Therefore, it would be of much benefit to produce low-cost oxygen monitoring devices that detect oxygen concentrations within this range; such sensors could be particularly useful as individual gas monitors for personnel working in confined spaces. A range of portable sensors have been developed to monitor oxygen concentrations, including potentiometric sensors (e.g., zirconia solid-electrolyte based ‘lambda sensors’) [7], and impedimetric sensors based on semiconducting metal-oxides (e.g., SnO_2_, TiO_2_, ZnO, and WO_3_) [8] or those based on conducting polymers [9]. Amperometric gas sensors for oxygen are also widely used due to their small size, low-cost, high stability, good sensitivity and selectivity, low power consumption and fast response times [10]. However, commercially-available amperometric sensors can suffer from issues with solvent evaporation, particularly in hot and dry environments [10]. One way to address this issue is to use non-volatile room temperature ionic liquids (RTILs) as solvents [11].

In this work, RTILs are used together with miniaturised, low-cost screen-printed electrode devices to detect oxygen at percent concentrations. RTILs are salts that are liquid at room temperature and possess properties such as low volatility, wide electrochemical windows, intrinsic conductivity, high polarity, good chemical and physical stability, tunability, high viscosity, and good solvation ability [12,13,14,15]. Screen-printed electrodes (SPEs) are devices where all three electrodes (working, counter, and reference) are printed in a small area on a planar substrate and have attracted much attention over the last decade as cheap, miniaturised platforms for electrochemical reactions and electroanalytical studies [16,17,18,19,20,21]. Various materials (e.g., carbon, platinum, gold, silver) have been made available as working electrodes, but carbon-based SPEs are, by far, the most widely investigated due to their low-cost, acceptable conductivity and their versatility for miniaturisation. Many of the mechanistic and analytical studies on SPEs have taken place in aqueous environments, and mimic closely the response of conventional electrodes such as edge plane pyrolytic graphite electrodes [22]. Typically, the electrodes are used without modification “as-is”, but strategies have also been explored to study surface pre-treatments, such as electrochemical activation [16,23], DMF soaking [23,24,25], NaOH soaking [23,26], ultrasonication [27], plasma treatment [28,29], UV-ozone treatment [23], and mechanical polishing [23,30,31,32], to improve the surface kinetics and/or analytical performance.

In addition to aqueous solvents, room-temperature ionic liquids (RTILs) have also been used together with SPEs for electroanalytical applications [14,31,33,34,35,36,37,38]. Gomis-Berenguer et al. [34] explored the electrochemical behaviour of various dissolved species (ferrocene, 1,4-benzoquinone, 1,4-diphenyl-9,10-anthraquinone, tetracyclone, and benzophenone-3) in the RTIL 1-hexyl-3-methylimidazolium hexafluorophosphate ([C_6_mim][PF_6_]) on homemade basal and edge screen-printed graphite electrodes (SPGEs). Benzophenone-3 was detected analytically, and the applicability of screen-printed platforms for electroanalytical measurements in RTILs was discussed [34]. The behaviour of various gases (e.g., oxygen [31,35,38,39], ammonia [14,40], chlorine [36], methylamine [37], and hydrogen chloride [37]) in RTILs on commercially-available SPEs has also been reported. The majority of these studies employed Pt or Au SPEs, but carbon-based SPEs from DropSens have also been used for ammonia [14] and oxygen detection [35]. In both cases, the C-SPEs were far inferior (i.e., higher LODs, large capacitive currents, more signal deterioration) compared to their Pt counterparts. Notably, for the case of oxygen reduction, the SPE material was found to promote the reaction of the imidazolium cation with electrogenerated superoxide, resulting in non-ideal voltammetry and electrode fouling over time [35]. Mechanical polishing of the Pt-SPE surface was later found to improve the response so that long-term continuous detection of oxygen (from 0.1% to 5% vol.) could be achieved [31].

This work will explore, for the first time, the short-term and long-term electrochemical behaviour of home-made edge-type screen-printed graphite electrodes (SPGEs) towards oxygen gas in RTILs, under ambient pressure and temperature conditions. Notably, these carbon-based surfaces are approximately 5–10 times cheaper than their platinum counterparts. A comparison will also be made with commercially-available carbon SPEs (C-SPEs) from DropSens, which have different ink formulations. The results will evaluate whether these electrodes are suitable to be used with RTIL solvents for low-cost gas sensing applications.

## 2. Experimental Section

### 2.1. Chemical Reagents

The RTILs 1-ethyl-3-methylimidazolium bis(trifluoromethylsulfonyl)imide ([C_2_mim][NTf_2_]), 1-butyl-3-methylimidazolium bis(trifluoromethylsulfonyl)imide ([C_4_mim][NTf_2_]), and *N*-butyl-*N*-methylpyrrolidinium bis(trifluoromethylsulfonyl)imide ([C_4_mpyrr][NTf_2_]) were purchased from IoLiTec Ionic Liquids Technologies GmbH (Salzstrasse, Heilbronn, Germany). The RTILs 1-hexyl-3-methylimidazolium trifluorotris(pentafluoroethyl)phosphate ([C_6_mim][FAP]), 1-butyl-3-methylimidazolium hexafluorophosphate ([C_4_mim][PF_6_]), and 1-butyl-3-methylimidazolium tetrafluroborate ([C_4_mim][BF_4_]), were purchased from Merck KGaA (Kilsyth, Victoria, Australia) at ultra-high purity electrochemical grade. All RTILs were used as received. Acetonitrile (MeCN, 99.8%, Sigma-Aldrich, Castle Hill, NSW, Australia), ethanol (EtOH, Sigma-Aldrich, 99%), and ultrapure water (with a resistivity of 18.2 MΩ·cm prepared by a Milli-Q laboratory water purification system (Millipore Pty Ltd., North Ryde, NSW, Australia) were used for rinsing the electrodes. High-purity oxygen gas (O_2_, >99.5%) and high-purity nitrogen gas (N_2_, >99.99%) were purchased from BOC gases (North Ryde, NSW, Australia). 

### 2.2. Materials

Edge-type screen-printed graphite electrodes (denoted as SPGEs in this paper) were fabricated in-house by Banks’s group [41,42,43], consisting of a graphite ink working electrode (3.1 mm diameter), a graphite ink counter electrode, and a silver/silver chloride quasi-reference electrode. Scanning electron microscopy (SEM) images of the SPGE working electrode are shown in Figure S1 of the supporting information. The SPEs were fabricated with the appropriate stencils using a DEK 248 screen-printing machine (DEK, Granby Industrial Estate, Weymouth, UK). First, a carbon-graphite ink formulation (product code C2000802P2; Gwent Electronic Materials Ltd., Mamhilad Park, Pontypool, UK) was screen-printed onto a polyester flexible film (Autostat, 250 μm thickness) and cured in a fan oven at 60 °C for 30 min. Next, a silver/silver chloride reference electrode was included by screen-printing Ag/AgCl paste (product code C2030812P3; Gwent Electronic Materials Ltd.) onto the polyester substrate and a second curing step was undertaken (60 °C for 30 min). Finally, a dielectric paste (product code D2070423D5; Gwent Electronic Materials Ltd.) was then printed onto the polyester substrate to cover the electrical connections. After a final curing step at 60 °C for 30 min the SPGEs were ready to be used. Dimensions of the overall device are 42 × 7.5 × 0.3 mm.

Commercially-available carbon screen-printed electrodes (denoted as C-SPEs in this paper) were purchased from DropSens (Llanera, Asturias, Spain), consisting of a C working electrode (4 mm diameter), a C counter electrode, and a silver quasi-reference electrode (code: DRP110), with overall dimensions of the device of 34 × 10 × 0.5 mm. The manufacturer does not disclose the exact formulations of the inks. A SEM image of the C-SPE working electrode can be found in reference [35]. To prevent excessive spreading of the RTIL on the electrode, a ca. 2 mm tall silicone barrier (Selleys Wet Area Silicone, purchased off-the-shelf) was constructed around the planar three-electrode cell on both devices, and allowed to set overnight. Care was taken to ensure that the silicone barrier did not cover the working, counter or reference electrodes. Contact angle measurements of the different RTIL used were characterized with a goniometer (KVS Instruments Ltd., Model: CAM 101, Höyläämötie, Helsinki, Finland). The amount of spread varied between the RTILs, with contact angles on the SPGE working electrode ranging from 14.5° to 45.3° for the six RTILs (see Table S1 in the Supporting Information), and correlated well with the saturated water content of the RTIL. The best surface “wetting” was observed for the more hydrophilic [C_4_mim][PF_6_] and [C_4_mim][BF_4_] RTILs, suggesting that the graphite working electrode surface is relatively hydrophilic, in accordance with a previous report [34].

### 2.3. Electrochemical Experiments

**Figure 1:**
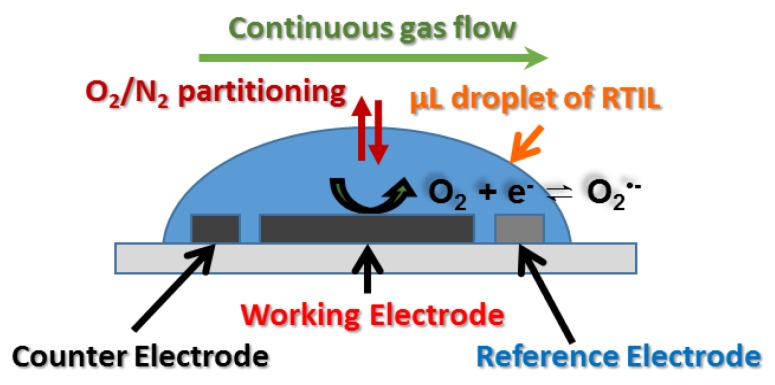
Illustration (not to scale) of the side-view of a microliter droplet of RTIL covering the working, counter, and reference electrodes of a screen-printed electrode device.

All electrochemical experiments were performed using a PGSTAT101 potentiostat (Eco-Chemie, Kanaalweg, Utrecht, The Netherlands) interfaced to a PC with NOVA 1.11.2 software. All experiments were carried out inside a custom-made aluminium Faraday cage to reduce ambient electromagnetic interferences, and at a temperature of 294 ± 1 K. A step potential of 2.5 mV and a scan rate of 10 mV·s^−1^ was used for cyclic voltammetry (CV) experiments, unless otherwise stated. Long-term chronoamperometry (LTCA) measurements were conducted with a sampling interval time of 2.5 s, and at a suitable overpotential determined from preliminary CV scans. The electrolyte was left under a flowing N_2_ environment for >2 h to allow any O_2_, moisture and other absorbed gases to be purged before the electrochemical measurements were commenced. After the introduction of oxygen, CV was conducted at 5 min intervals to ensure that the gas was fully saturated, and that the response was stable. Ca. 15 min was typically sufficient to saturate 20/30 μL of the neat RTIL. A side-view of a droplet of RTIL on a screen-printed electrode, and the sensing processes, are shown in Figure 1. The (O_2_ and N_2_) gases flow continuously over the RTIL, and are partitioned into the RTIL at the gas-ionic liquid interface. The analyte gas (oxygen) then diffuses towards the electrode, where it is reduced upon the application of a sufficiently negative potential.

Twenty microlitres of RTIL was used for the SPGE, while 30 μL was used for the (slightly larger) C-SPE to cover the working, counter and reference electrodes, before the device was inserted using a rubber stopper into a modified glass cell, as described previously [35]. Aluminium wires were carefully secured with a clip onto the screen-printed carbon leads for the SPGEs (since soldering onto the connections was not possible), and soldered onto the C-SPE connections to allow for easy interfacing to the potentiostat. Due to the use of an unstable quasi-reference, some drift in the potentials was observed during the O_2_ experiments. All the peak currents were obtained after first correcting for the background currents by extrapolating a linear line from the non-Faradaic region of the scan. For CVs obtained where the peak current/potential was not clear, the peak potential was first estimated by analysing the derivatives of the CVs (i.e., finding the maxima of δ*I*/δ*E*), to enable consistent extraction of peak currents for use in the calibration plots, and peak potentials for estimating the peak-to-peak separations (Δ*E*_p_). 

### 2.4. Gas Mixing Setup

Oxygen was diluted with N_2_ gas using a gas mixing system detailed in our previous work [35], and the concentrations were controlled based on the ratios of flow rates in the two digital flow controllers (Cole-Parmer, Victoria Ave, Chatswood, NSW, Australia). For CV experiments, a concentration range between 10–100% vol. for O_2_ was investigated. For LTCA measurements, two concentration ranges were employed, termed “low concentration” (0.1–20% vol. O_2_) and “high concentration” (1–100% vol. O_2_). For experiments within the “low concentration” range, the N_2_ flow rate was fixed at 1000 sccm, and the O_2_ flow rate varied. For the “high concentration” range, the N_2_ flow rate was fixed at 180 sccm, while varying the O_2_ flow rate. For LTCA, the initial response was allowed to stabilize (under N_2_) for ca. 30 min before commencing with the introduction of O_2_ gas. Adjusting of the flow rates was automated using a computer software controller developed in-house [44]. It is noted that the detection of ammonia gas on the same surfaces was also studied, but both carbon-based surfaces proved to be unsuitable for accurate detection of the gas due to the lack of a clear oxidation peak (see Supporting Information). This is not the case on both platinum and gold SPEs, where clear peaks are observed for ammonia oxidation in RTILs [14,40].

## 3. Results and Discussion

### 3.1. Cyclic Voltammetry

**Figure 2:**
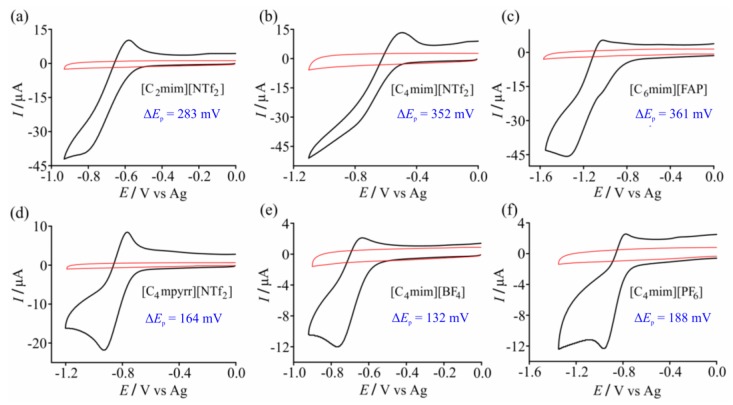
CVs (10 mV·s^−1^) for the reduction of 100% vol. O_2_ on SPGEs in 6 different RTILs: (**a**) [C_2_mim][NTf_2_], (**b**) [C_4_mim][NTf_2_], (**c**) [C_6_mim][FAP], (**d**) [C_4_mpyrr][NTf_2_], (**e**) [C_4_mim][BF_4_], and (**f**) [C_4_mim][PF_6_]. The peak-to-peak potentials, Δ*E*_p_, are indicated in blue. Blank CVs (in the absence of oxygen) are shown as red solid lines.

Six common RTILs were initially examined as solvents for oxygen (O_2_) reduction on the edge-type screen-printed graphite electrodes (SPGEs), in order to determine two RTILs to be used for further analytical studies. In RTILs, O_2_ is reduced to superoxide in a chemically reversible one electron reduction mechanism, as described in Equation (1) [45,46,47]. Figure 2 shows cyclic voltammetry (CV) for the reduction of 100% vol. O_2_ at 10 mV·s^−1^ in the RTILs (a) [C_2_mim][NTf_2_], (b) [C_4_mim][NTf_2_], (c) [C_6_mim][FAP], (d) [C_4_mpyrr][NTf_2_], (e) [C_4_mim][BF_4_], and (f) [C_4_mim][PF_6_]. The blank CV in the absence of oxygen (red line) is also shown in the figure. As can be seen, the blank response in all six RTILs is flat and featureless on the SPGE. This is far superior to that observed on commercially-available carbon screen-printed electrodes (C-SPEs) from DropSens, where there was significantly more capacitive current and additional voltammetric features observed, particularly in the negative potential window [35].

(1) $$O_{2\;\left(g\right)}+e^{-}⇌O_{2\;\left(\mathrm{RTIL}\right)}^{\;\cdot -}$$

The reduction of oxygen is peak-shaped in Figure 1 (at 10 mV∙s^−1^) in four of the RTILs, but is steady-state in [C_2_mim][NTf_2_] and [C_4_mim][NTf_2_]. For comparison, the CVs in all RTILs are typically peak-shaped on conventional Pt macrodisk electrodes, [35] but are usually steady-state on Pt microdisk electrodes [48]. The values of the peak-to-peak separations (Δ*E*_p_) are given in the figure, with estimations made where the potential of the reduction peak was not obvious (see details in the Experimental section). The smallest (Δ*E*_p_) was observed in the RTILs [C_4_mpyrr][NTf_2_], [C_4_mim][BF_4_], and [C_4_mim][PF_6_], which is fairly consistent with that observed in our previous work on a conventional Pt macrodisk electrode [35]. Δ*E*_p_ increases significantly at higher scan rates in all RTILs (see Figure S2 and Table S2 in the supporting information), and significant Ohmic drop is observed at scan rates above 100 mV·s^−1^. As a result, the optimum scan rate chosen for presentation of the CVs in this work is 10 mV∙s^−1^. The CVs for oxygen reduction on DropSens C-SPEs were also studied for comparison (see Figure S3 in the supporting information). Much larger background capacitive currents were observed on the C-SPE compared to the SPGE (particularly obvious at higher scan rates), possibly due to differences in the ink formulation and their compatibility with the RTIL solvents. Interestingly, Δ*E*_p_ increased more dramatically for [C_4_mim][PF_6_] on the C-SPE compared to the SPGE, and the opposite was true for [C_2_mim][NTf_2_]. This could be due to the different wetting characteristics of the RTIL on the electrode surface (with the C-SPE being less hydrophilic than the SPGE), resulting in faster kinetics for the oxygen/superoxide redox couple in [C_4_mim][PF_6_] on the SPGE.

**Figure 3:**
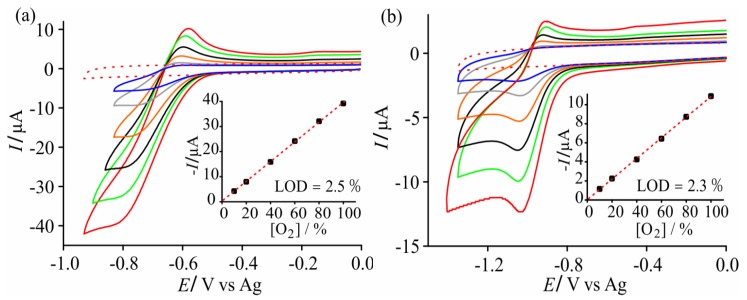
CVs at different oxygen concentrations (10–100% vol.) in (**a**) [C_2_mim][NTf_2_] and (**b**) [C_4_mim][PF_6_] at 10 mV∙s^−1^ on a SPGE. Blank CVs (in the absence of oxygen) are shown as red dashed lines. The insets show the calibration plots and lines of best fit, with the LOD values (based on three standard deviations of the line).

In order to study the analytical properties of these electrodes, CV was carried out at different concentrations of oxygen. Figure 3 shows CVs at 10 mV∙s^−1^ for 10–100% vol. O_2_ in (a) [C_2_mim][NTf_2_] and (b) [C_4_mim][PF_6_], and the corresponding calibration graphs in the insets. These two ionic liquids were chosen as they are the lowest and highest viscosity electrolytes out of the six RTILs studied, and gave the most stable and reproducible results. CVs for the remaining RTILs at 10–100% vol. O_2_ are shown in Figure S4 of the supporting information. A slanted limiting current plateau was observed in [C_4_mim][NTf_2_], and potential shifting was the most significant in [C_4_mpyrr][NTf_2_]. The behaviour in the [C_6_mim][FAP] ionic liquid deviates the most from an ideal CV shape, and became worse at lower concentrations such that stable CVs could not be collected. Unusual behaviour in RTILs with [FAP]^−^ anions has also been well reported in the literature [49,50]. Limits of detection (LODs, calculated from three standard deviations of the slope of the line of best fit) were 2.5% and 2.3% vol. O_2_ in [C_2_mim][NTf_2_] and [C_4_mim][PF_6_], respectively, and could likely be reduced further if a lower concentration range is studied. The only RTIL that gave a lower LOD than these two RTILs was [C_4_mim][BF_4_] (see Figure S4), although it is noted that this RTIL is fully miscible with water [51] and may not be suitable for sensing of gases in high-humidity environments. 

### 3.2. Chronoamperometry

**Figure 4:**
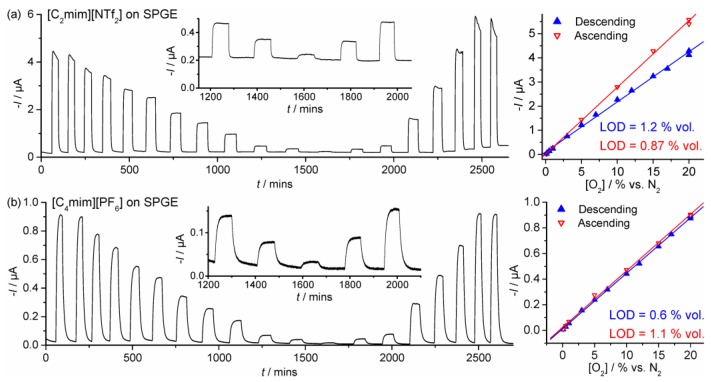
LTCA for different concentrations of oxygen in (**a**) [C_2_mim][NTf_2_] and (**b**) [C_4_mim][PF_6_] on a SPGE for 20, 20, 17, 15, 12, 10, 7, 5, 3, 1, 0.5, 0.1, 0.5, 1, 5, 10, 15, 20, and 20% vol. O_2_ alternating with periods of N_2_ purging. Potentials were held at −0.9 V and −1.2 V, respectively. The inset shows a plot zoomed in at the lowest concentrations (1, 0.5, 0.1, 0.5, and 1% vol. O_2_). The respective calibration plots for the descending and ascending concentrations are shown on the right.

Long-term chronoamperometry (LTCA) was performed to test the viability of the SPGEs for continuous monitoring of oxygen. It is noted that this is a very harsh technique due to the constant build-up of superoxide during constant potential biasing, and the long experimental timescale employed (ca. 20–50 h) due to slow partitioning/equilibration of the gas into the (relatively large) 20 or 30 μL droplet of RTIL employed. Figure 4 shows LTCA for O_2_ reduction in (a) [C_2_mim][NTf_2_] and (b) [C_4_mim][PF_6_] at 20 to 0.1 to 20% vol. O_2_, (the “low concentration” range) alternating with periods of nitrogen purging (to observe the stability of the baseline). In [C_2_mim][NTf_2_], the current response behaviour is as expected for a reduction process, but currents are unstable and do not reach a plateau at concentrations above ca. 10% vol. O_2_. Additionally, the calibration graphs from the (initial) descending and (subsequent) ascending concentrations have different gradients, which indicates that this RTIL may not be suitable for long-term sensing. However, the behaviour in [C_4_mim][PF_6_] was significantly more stable, where the response reaches a stable plateau, and the ascending and descending concentration calibration plots were almost identical. This is far superior to the behaviour on Pt-SPEs in both [C_2_mim][NTf_2_] and [C_4_mpyrr][NTf_2_] that we have reported previously, where calibration graphs could not even be achieved [31]. The reproducibility of the current is good, as observed by recording very similar currents for the two repeated 20% O_2_ data points on both the descending and ascending plots (4 data points in total on Figure 4b). Error bars would likely be very small in this case.

**Figure 5:**
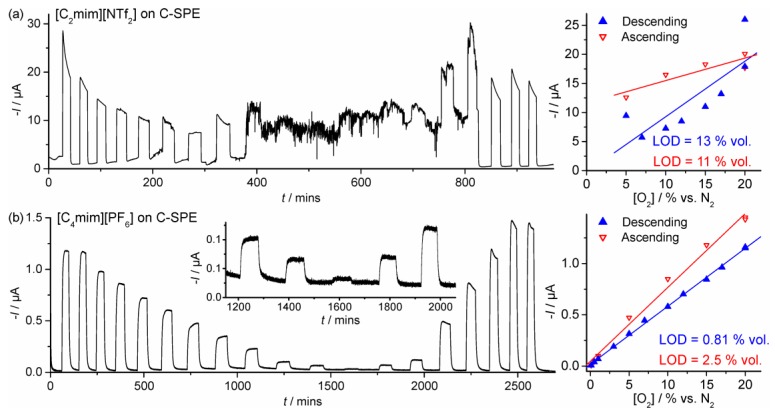
(**Left**) LTCA for different concentrations of oxygen in (**a**) [C_2_mim][NTf_2_] and (**b**) [C_4_mim][PF_6_] on a DropSens C-SPE for 20, 20, 17, 15, 12, 10, 7, 5, 3, 1, 0.5, 0.1, 0.5, 1, 5, 10, 15, 20, and 20% vol. O_2_ alternating with periods of N_2_ purging, conducted at 0.15 to 0.20 V negative to the O_2_ reduction peak potentials. The inset shows a plot zoomed into the lowest concentrations (1, 0.5, 0.1, 0.5, and 1% vol. O_2_). (**Right**) Calibration plots for the initial descending and subsequent ascending change in O_2_ concentrations.

Equivalent experiments were performed for O_2_ reduction on the DropSens C-SPE in [C_2_mim][NTf_2_] and [C_4_mim][PF_6_] (see Figure 5), but the currents were significantly more unstable and erratic compared to the SPGE. The equations of the calibration graphs and LODs (3σ) are given in Table 1. Sensitivities (gradients) are higher in [C_2_mim][NTf_2_] compared to [C_4_mim][PF_6_] probably due to the lower viscosity of [C_2_mim][NTf_2_] and higher O_2_ solubility. Sensitivities are also higher on the C-SPE compared to the SPGE due to the larger surface area of the C-SPE. LODs are relatively high in [C_2_mim][NTf_2_] (1.2% and 13% vol. O_2_ on the SPGE and C-SPE, respectively), but are below 1% vol. O_2_ in [C_4_mim][PF_6_] on both electrodes. The SPGE likely gives a better sensing performance due to the different materials used in the screen-printed pastes which are less prone to attack by the electrogenerated superoxide [25] compared to the commercial C-SPE. Therefore, it can be concluded that the SPGE is a substantially better surface for long-term continuous monitoring of oxygen (in the lower concentration range), and that the RTIL [C_4_mim][PF_6_] shows superior behaviour to [C_2_mim][NTf_2_].

Experiments at O_2_ concentrations up to 100% vol. on the SPGE were also carried out to see if these electrodes perform well at the “high concentration” range (1–100% vol. O_2_). Li and Compton [52] recognise that amperometric sensing at high oxygen concentrations is difficult and “needs innovation”, and so it would be useful to assess the suitability of these low-cost screen-printed electrode platforms for the detection of oxygen gas at the high concentration limit. Figure S5 in the supporting information shows LTCA performed at 1–100% vol. O_2_ in [C_2_mim][NTf_2_] and [C_4_mim][PF_6_] on the SPGE and [C_4_mim][PF_6_] on the C-SPE, using the same electrodes that had already been subjected to LTCA experiments at the low concentration range. Interestingly, the responses were quite reasonable and did not significantly deteriorate, although ideal current plateaus were not observed on all electrodes, and a drift in the baseline current was observed in [C_2_mim][NTf_2_]. Calibration graphs were relatively linear, and the analytical parameters for all calibration graphs are given in Table S4 of the supporting information.

Response times can also be estimated from the LTCA experiments in Figure 4 and Figure 5. At 20% vol. O_2_, 90% response times (*t*_90_) are ca. 2.7 min and 13 min in [C_2_mim][NTf_2_] and [C_4_mim][PF_6_], respectively. The equivalent *t*_90_ responses for 20% vol. O_2_ on the C-SPE are ca. 0.6 mins and 7.3 min in [C_2_mim][NTf_2_] and [C_4_mim][PF_6_], respectively. The shorter response times for [C_2_mim][NTf_2_] are likely due to the lower viscosity of the RTIL compared to [C_4_mim][PF_6_], although the different volumes and amount of spreading of the RTIL may also have an effect. We note here that response times can be reduced significantly by employing thinner layers of RTIL, although this is not the focus of the present work.

**Figure 6:**
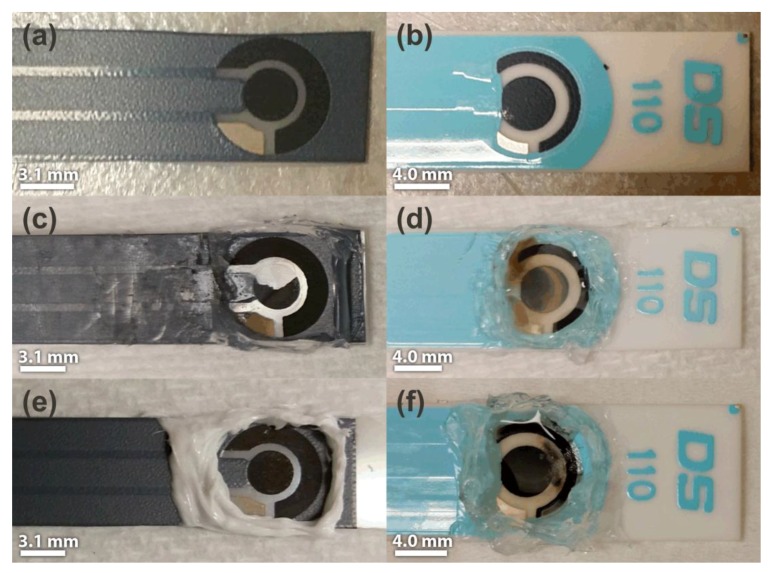
Photographs of the electrodes before and after being subjected to long-term oxygen sensing experiments using LTCA: (**a**) unused SPGE, (**b**) unused DropSens C-SPE, (**c**) SPGE with [C_2_mim][NTf_2_], (**d**) DropSens C-SPE with [C_2_mim][NTf_2_], (**e**) SPGE with [C_4_mim][PF_6_], and (**f**) DropSens C-SPE with [C_4_mim][PF_6_]. Scale bars indicate the diameter of the working electrode.

After LTCA experiments, some visible degradation of the screen-printed materials was observed. Figure 6 shows photographs of (left) home-made SPGEs and (right) DropSens C-SPEs SPEs, before (Figure 6a,b), and after LTCA experiments in [C_2_mim][NTf_2_] (Figure 6c,d) and [C_4_mim][PF_6_] (Figure 6e,f). In [C_2_mim][NTf_2_], a large piece of the SPGE working electrode broke off, leaving the underlying substrate exposed, and the reference and counter electrodes were also altered (Figure 6c). In the same RTIL on the C-SPE (Figure 6d), browning of the RTIL was observed, suggesting that some of the screen-printed material was dispersed into the RTIL, but that the working electrode did not completely break away completely from the substrate (i.e., there was good adhesion). The significantly higher LTCA currents in [C_2_mim][NTf_2_] on the C-SPE also support the breaking-up of the electrode surface during the experiments. In [C_4_mim][PF_6_], the SPGE was unaffected by the harsh long-term experiments (Figure 6e), but some of the screen-printed materials became dispersed in the RTIL on the DropSens C-SPE (Figure 6f). A possible reason for the more drastic degradation of the screen-printed surfaces in [C_2_mim][NTf_2_] compared to [C_4_mim][PF_6_] is that the current was ca. four times higher in [C_2_mim][NTf_2_], resulting in more build-up of products at the working and counter electrodes. A summary of these observations is given in Table 2. These observations suggest that the SPGE and [C_4_mim][PF_6_] is the best electrode/RTIL combination for long-term continuous oxygen monitoring applications.

## 4. Conclusions

Low-cost screen-printed graphite electrodes (SPGEs) with RTIL solvents have been investigated as devices for oxygen detection. The CVs appeared to show a typical reversible one-electron reduction to superoxide, but with significant Ohmic drop contributions at scan rates above 100 mV∙s^−1^. LTCA results revealed varying responses on the SPGEs vs. C-SPEs, with the overall sensing performance being superior on the SPGEs. When different RTILs were employed, different stabilities and amounts of degradation were observed—such as the breaking off or dispersion of the working electrode material into the RTIL. This suggests that the choice of RTIL is very important for long-term gas sensing experiments. Overall, SPGEs with [C_4_mim][PF_6_] gave the best analytical responses; this combination appears to be a viable RTIL/SPE-based system for mass-producible, miniaturised, ultralow-cost O_2_ gas sensors. 

## Figures and Tables

**Table 1:** Analytical parameters obtained from calibration graphs from LTCA experiments for the reduction of oxygen in the two chosen RTILs, on both the screen-printed graphite electrode (SPGE) and carbon screen-printed electrode (C-SPE) surfaces. Equations of the linear best fit, limits of detection (LODs), and *R*^2^ values of the calibration plots obtained at the low concentration range (0.1–20%, descending) are shown (see Figure 3 and Figure 4). The full set of data from all SPE/RTIL combinations recorded is shown in Table S4 of the Supporting Information.

Electrode	RTIL	[O_2_] Range	Order	Equation of Linear Best Fit	LOD	*R*^2^
		/% vol.		*I*/A, [O_2_]/% vol.	/% vol.	
SPGE	[C_2_mim][NTf_2_]	0.1–20	Descending	*I* = 2.07 × 10^−7^[O_2_] + 1.02 × 10^−7^	1.2	0.997
C-SPE			Descending	*I* = 9.55 × 10^−7^[O_2_] − 2.72 × 10^−7^	13	0.663
SPGE	[C_4_mim][PF_6_]	0.1–20	Descending	*I* = 4.33 × 10^−8^[O_2_] + 1.22 × 10^−8^	0.60	0.999
C-SPE			Descending	*I* = 5.65 × 10^−8^[O_2_] + 1.85 × 10^−8^	0.81	0.999

**Table 2:** Overview of physical observations of the SPGEs and C-SPEs following short-term cyclic voltammetry experiments, and harsher long-term chronoamperometry experiments.

Electrode	RTIL	Comments on Short-Term Stability (After CV Experiments)	Comments on Long-Term Stability (after LTCA Experiments)
SPGE	[C_2_mim][NTf_2_]	Stable, no deterioration observed	Working electrode broke off in a large chunk
C-SPE	[C_2_mim][NTf_2_]	Stable, no deterioration observed. Some darkening of RTIL	Significant darkening of RTIL, and working and reference electrodes tarnished.
SPGE	[C_4_mim][PF_6_]	Stable, no deterioration observed	Stable, no deterioration observed
C-SPE	[C_4_mim][PF_6_]	Stable, no deterioration observed	Darkening of RTIL observed

## Supplementary Materials

The following are available online at www.mdpi.com/1424-8220/17/12/2734/s1; **Figure S1.** Scanning electron microscopy (SEM) images of the home-made SPGE used in this work, at two different magnifications. These electrodes are heterogeneous and are made up of conductive graphite and carbon black particles held together by a polymeric binder. They are relatively rough but are not porous. These electrodes do have a high proportion of edge plane sites/defects and hence that is the reason they give rise to electrochemically useful signatures; **Figure S2.** CVs. at different scan rates (10, 50, and 100 mV∙s^−1^) for the reduction of 100% vol. oxygen on a SPGE in the six RTILs used in this study; **Figure S3.** CVs. at different scan rates (10, 50, and 100 mV∙s^−1^) on DropSens C-SPEs for 100% vol. O_2_ in (**a**) [C_2_mim][NTf_2_] and (**b**) [C_4_mim][PF_6_]; **Figure S4.** CVs. at 10 mV∙s^−1^ on a SPGE for different concentrations of oxygen (10–100% vol.) in the other four RTILs (not shown in the main text). The inset shows the respective calibration plots with the *R*^2^ and limit of detection (LOD) values; **Figure S5.** (**Left**) LTCA for different concentrations of oxygen in (**a**) [C_2_mim][NTf_2_] on a SPGE, (**b**) [C_4_mim][PF_6_] a SPGE, and (**c**) [C_4_mim][PF_6_] on a DropSens C-SPE for 1, 5, 10, 20, 40, 60, 80, 100, 80, 60, 40, 20, 10, 5, and 1% vol. O_2_ alternating with periods of N_2_ purging, performed after running the calibration experiments at the lower (0.1–20% vol. O_2_) concentration range on the same system, conducted at 0.15 to 0.20 V negative to the O_2_ reduction peak potentials. LTCA for [C_2_mim][NTf_2_] on DropSens C-SPE were not conducted as the response had deteriorated during experiments at the lower concentration range. (**Right**) Calibration plots for the initial descending and subsequent ascending change in O_2_ concentrations; **Figure S6.** LTCA for different concentrations of oxygen in [C_4_mpyrr][NTf_2_] on (a) a SPGE and (b) a DropSens C-SPE for 20, 20, 17, 15, 12, 10, 7, 5, 3,1,0.5, 0.1, 0.5, 1, 3, 5, 7, 10, 12, 15, 17, 20, and 20% vol. O_2_ alternating with periods of N_2_ purging, conducted at 0.15 to 0.20 V negative to the O_2_ reduction peak potentials. The inset shows a plot zoomed into the lowest concentrations (1, 0.5, 0.1, 0.5, 1% vol. O_2_). The respective calibration plots for the initial descending and subsequent ascending change in O_2_ concentrations are shown on the right, **Figure S7.** CVs. at 10 mV∙s^−1^ for 500 ppm NH_3_ oxidation in (**a**) [C_2_mim][NTf_2_] and (**b**) [C_4_mim][PF_6_] on a SPGE. Blank CVs. (in the absence of ammonia) are shown as red dashed lines; **Figure S8.** CVs. at different scan rates (10, 50, and 100 mVs^−1^) for 500 ppm NH_3_ in (**a**) [C_2_mim][NTf_2_] and (**b**) [C_4_mim][PF_6_] on a SPGE; **Figure S9.** LTCA for 10–100 ppm NH_3_, alternating with periods of nitrogen purging, in [C_2_mim][NTf_2_] RTIL on a SPGE. The potential was biased at +1.25 V. Red arrows indicate the addition of ammonia (100, 80, 60, 40, 20, and 10 ppm) into the cell. The inset shows the corresponding calibration plot and LOD value. Currents were measured from the spike to the steady-state (or maximum) current value; **Figure S10.** LTCA for 10–100 ppm NH_3_ in [C_2_mim][NTf_2_] on a DropSens C-SPE. The potential was biased at +1.25 V vs. Ag, and the cell was flushed with nitrogen between each NH_3_ concentration change; **Figure S11.** PSCA for 10–100 ppm NH_3_ on a SPGE in (**a**) [C_2_mim][NTf_2_] and (**b**) [C_4_mim][PF_6_]. The potential was stepped from 0.0 V to +1.25 V and monitored for 10 seconds, although the first three seconds are shown in the figure for clarity. The blank response in the absence of ammonia is shown as the red dotted line. Currents were measured at a fixed time of 2.0 seconds after the potential step. The insets show the (background subtracted) calibration plots and LOD values; **Figure S12.** PSCA for 10–100 ppm NH_3_ in (a) [C_2_mim][NTf_2_] and (b) [C_4_mim][PF_6_] RTILs on a DropSens C-SPE. The potential was stepped from 0 V to +1.25 V and measured for 10 seconds (only the first three and six seconds, respectively, are shown in the figure for clarity). Currents for the calibration graphs were measured at 2 s; **Table S1.** Contact angle (*θ*) measurements for six different RTILs (1 μL volume) dropcast on a SPGE WE surface and on the SPGE polymer mask. Also shown are the measured saturated water contents of the RTILs, from O’Mahony et al.; **Table S2.** Viscosity, *η*, of the six RTILs used in this study, and peak-to-peak separations (Δ*E*_p_) measured for the oxygen/superoxide redox couple on the SPGE and C-SPE at scan rates of 10, 50, and 100 mV∙s^−1^ for 100% vol. O_2_; **Table S3.** Peak-to-peak separations (Δ*E*_p_) for the oxygen/superoxide redox couple on the SPGE at scan rates of 10, 50, and 100 mV∙s^−1^ at 10–100% O_2_ in the six different RTILs used in this study; **Table S4.** Analytical parameters obtained from calibration graphs from LTCA experiments for the reduction of oxygen in the six RTILs, on both the screen-printed graphite (SPGE) and carbon screen-printed electrode (C-SPE) surfaces. Equations of the linear best fit, limits of detection (LODs), and *R*^2^ values of the calibration plots obtained at the lower (0.1–20%) and higher (1.1–100%) O_2_ concentration ranges (see Figure 3 in the main text, and Figures S4, S5, and S6).
