# Supplementary material for: Screen-Printed Graphite Electrodes as Low-Cost Devices for Oxygen Gas Detection in Room-Temperature Ionic Liquids
Source: Sensors (Basel). 2017 Nov 26;17(12):2734. doi: 10.3390/s17122734 (PMC5751384; doi:10.3390/s17122734)
Supplement: Supplementary file 1 [file sensors-17-02734-s001.pdf]

Supporting Information for:

Screen-Printed Graphite Electrodes as Low-Cost Devices for Oxygen Gas Detection in Room-Temperature Ionic Liquids

Junqiao Lee [1], Ghulam Hussain [1], Craig E. Banks [2] and Debbie S. Silvester [1,*]

[1] Curtin Institute for Functional Molecules and Interfaces & Department of Chemistry, Curtin University, GPO Box U1987, Perth, WA 6845, Australia; juniaiko@gmail.com (J.L.); ghulam.hussain1985@gmail.com (G.H.);
[2] Faculty of Science and Engineering, Manchester Metropolitan University, Chester Street, Manchester M1 5GD, UK; C.Banks@mmu.ac.uk
* Correspondence: d.silvester-dean@curtin.edu.au; Tel.: +61-08-9266-7148; Fax: +61-08-9266-2300

Table S1. Contact angle (Θ) measurements for six different RTILs (1 µL volume) dropcast on a SPGE WE surface and on the SPGE polymer mask. The measured saturated water contents of the RTILs, from O'Mahony et al.[1] are also shown.

	On SPGE WE	On SPGE mask	Saturated H_2O content
RTIL	Θ/º	Θ/º	/ ppm
[C_6mim][FAP]	14.5 ± 0.1	3.3 ± 0.3	3,068
[C_4mim][NTf_2]	20.7 ± 0.2	6.5 ± 0.8	5,680
[C_4mpyrr][NTf_2]	27.0 ± 0.5	26.0 ± 0.8	11,407
[C_2mim][NTf_2]	26.7 ± 0.2	35.7 ± 0.4	19,940
[C_4mim][PF_6]	34.7 ± 0.7	45.3 ± 0.6	24,194
[C_4mim][BF_4]	45.3 ± 0.3	47.5 ± 0.3	miscible

[1] O'Mahony, A.M.; Silvester, D.S.; Aldous, L.; Hardacre, C.; Compton, R.G. *J. Chem. Eng. Data*, 2008, 53(12), 2884-2891.

Table S2. Viscosity, η, of the six RTILs used in this study, and peak-to-peak separations (ΔE_P) measured for the oxygen/superoxide redox couple on the SPGE and C-SPE at scan rates of 10, 50, and 100 mV·s^{-1} for 100% vol. O_2.

		ΔE_P/mV		
On SPGE	η §/cP	ν/mV·s^{-1}		
RTIL	at 293 K	10	50	100
[C_2mim][NTf_2]	34	283	393	505
[C_4mim][NTf_2]	52	352	606	777
[C_6mim][FAP]	74	361	601	773
[C_4mpyrr][NTf_2]	89	164	298	398
[C_4mim][BF_4]	112	132	188	247
[C_4mim][PF_6]	371	188	261	354
On C-SPE				
[C_2mim][NTf_2]	34	242	332	422
[C_4mim][PF_6]	371	142	359	557

§ Barrosse-Antle, L.E.; Bond, A.M.; Compton, R.G.; O'Mahony, A.M.; Rogers, E.I.; Silvester, D.S. *Chem. Asian J.* 2010, 5, 202–230.

Table S3. Peak-to-peak separations (ΔE_p) for the oxygen/superoxide redox couple on the SPGE at scan rates of 10, 50, and 100 mV·s^{-1} at 10–100% O_2 in the six different RTILs used in this study.

	ΔE_p/V					
Scan rate: 10 mVs^{-1}	[O_2]/vol%					
RTIL	10	20	40	60	80	100
[C_2mim][NTf_2]	(190)	(151)	(197)	(226)	257	283
[C_4mim][NTf_2]	(264)	252	360	(329)	(344)	(352)
[C_6mim][FAP]		—— N.A. ——				601
[C_4mpyrr][NTf_2]	(105)	98	120	134	154	164
[C_4mim][BF_4]	(151)	(159)	115	117	127	132
[C_4mim][PF_6]	(190)	117	112	127	139	188
Scan rate: 50 mVs^{-1}	[O_2]/vol%					
RTIL	10	20	40	60	80	100
[C_2mim][NTf_2]	137	173	229	286	352	393
[C_4mim][NTf_2]	264	252	360	329	344	352
[C_6mim][FAP]		—— N.A. ——				361
[C_4mpyrr][NTf_2]	142	154	193	237	278	298
[C_4mim][BF_4]	117	137	142	156	176	188
[C_4mim][PF_6]	(208)	168	176	195	222	261
Scan rate: 100 mV·s^{-1}	[O_2]/vol%					
RTIL	10	20	40	60	80	100
[C_2mim][NTf_2]	198	222	271	359	430	505
[C_4mim][NTf_2]	359	486	688	734	793	777
[C_6mim][FAP]		—— N.A. ——				773
[C_4mpyrr][NTf_2]	198	222	264	325	376	398
[C_4mim][BF_4]	146	173	188	205	227	247
[C_4mim][PF_6]	283	234	247	273	305	354

Numbers in brackets ( ) = estimated values, since the peak potentials from CVs were unclear.

Table S4. Analytical parameters obtained from calibration graphs from LTCA experiments for the reduction of oxygen in the six RTILs, on both the screen-printed graphite (SPGE) and carbon screen-printed electrode (C-SPE) surfaces. Equations of the linear best fit, limits of detection (LODs), and R^2 values of the calibration plots obtained at the lower (0.1–20%) and higher (1.1–100%) O_2 concentration ranges (see Figure 3 in the main text, and Figures S4, S5, and S6).

SPE	RTIL	$[O_2]$ range /vol%	Order	Equation of linear best fit I/A, $[O_2]$/vol%	LOD /vol%	R^2
SPGE	$[C_2mim][NTf_2]$	0.1–20	Descending	$I = 2.07 \times 10^{-7}[O_2] + 1.02 \times 10^{-7}$	1.2	0.997
			Ascending	$I = 2.76 \times 10^{-7}[O_2] + 2.33 \times 10^{-8}$	0.87	0.999
C-SPE			Descending	$I = 9.55 \times 10^{-7}[O_2] - 2.72 \times 10^{-7}$	13	0.663
			Ascending	$I = 3.86 \times 10^{-7}[O_2] + 1.16 \times 10^{-5}$	11	0.809
SPGE	$[C_4mim][PF_6]$	0.1–20	Descending	$I = 4.33 \times 10^{-8}[O_2] + 1.22 \times 10^{-8}$	0.60	0.999
			Ascending	$I = 4.41 \times 10^{-8}[O_2] + 2.28 \times 10^{-8}$	1.1	0.998
C-SPE			Descending	$I = 5.65 \times 10^{-8}[O_2] + 1.85 \times 10^{-8}$	0.81	0.999
			Ascending	$I = 7.19 \times 10^{-8}[O_2] + 4.82 \times 10^{-8}$	2.5	0.992
SPGE	$[C_2mim][NTf_2]$	1.1–100	Ascending	$I = 2.64 \times 10^{-7}[O_2] - 1.30 \times 10^{-7}$	2.4	0.999
			Descending	$I = 2.63 \times 10^{-7}[O_2] - 3.06 \times 10^{-7}$	3.8	0.999
C-SPE			Ascending	—— N.A. ——		
			Descending	—— N.A. ——		
SPGE	$[C_4mim][PF_6]$	1.1-100	Ascending	$I = 2.01 \times 10^{-8}[O_2] + 3.46 \times 10^{-8}$	13	0.988
			Descending	$I = 1.89 \times 10^{-8}[O_2] - 4.34 \times 10^{-8}$	11	0.992
C-SPE			Ascending	$I = 6.30 \times 10^{-8}[O_2] + 1.87 \times 10^{-7}$	7.9	0.996
			Descending	$I = 6.45 \times 10^{-8}[O_2] - 9.40 \times 10^{-8}$	11	0.992
SPGE	$[C_4mpyrr][NTf_2]$	0.1–20	Descending	$I = 4.69 \times 10^{-8}[O_2] + 1.83 \times 10^{-8}$	0.93	0.998
			Ascending	$I = 5.50 \times 10^{-8}[O_2] + 7.45 \times 10^{-9}$	0.27	0.999
C-SPE			Descending	$I = 6.47 \times 10^{-8}[O_2] + 3.36 \times 10^{-8}$	1.7	0.995
			Ascending	$I = 6.26 \times 10^{-8}[O_2]+7.64 \times 10^{-8}$	3.5	0.984

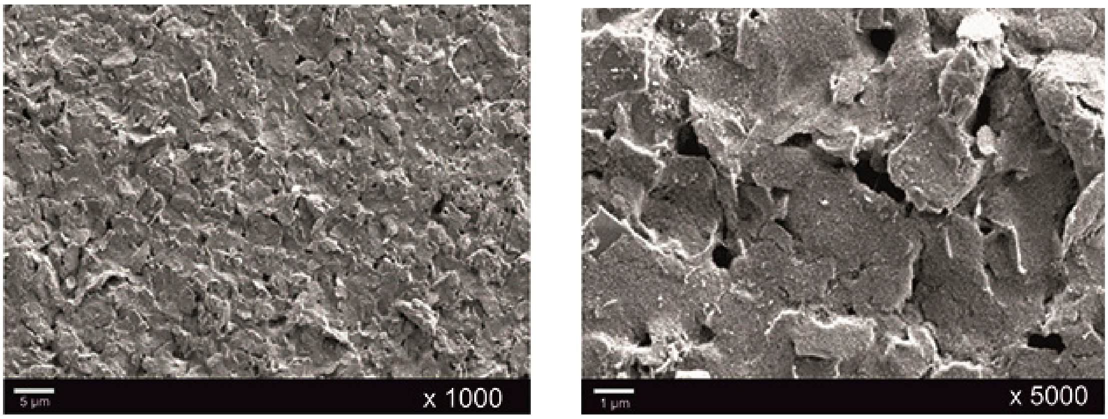

Figure S1. Scanning electron microscopy (SEM) images of the home-made SPGE used in this work, at two different magnifications. These electrodes are heterogeneous and are made up of conductive graphite and carbon black particles held together by a polymeric binder. They are relatively rough, but are not porous. These electrodes do have a high proportion of edge plane sites/defects and, hence, that is the reason they give rise to electrochemically useful signatures.

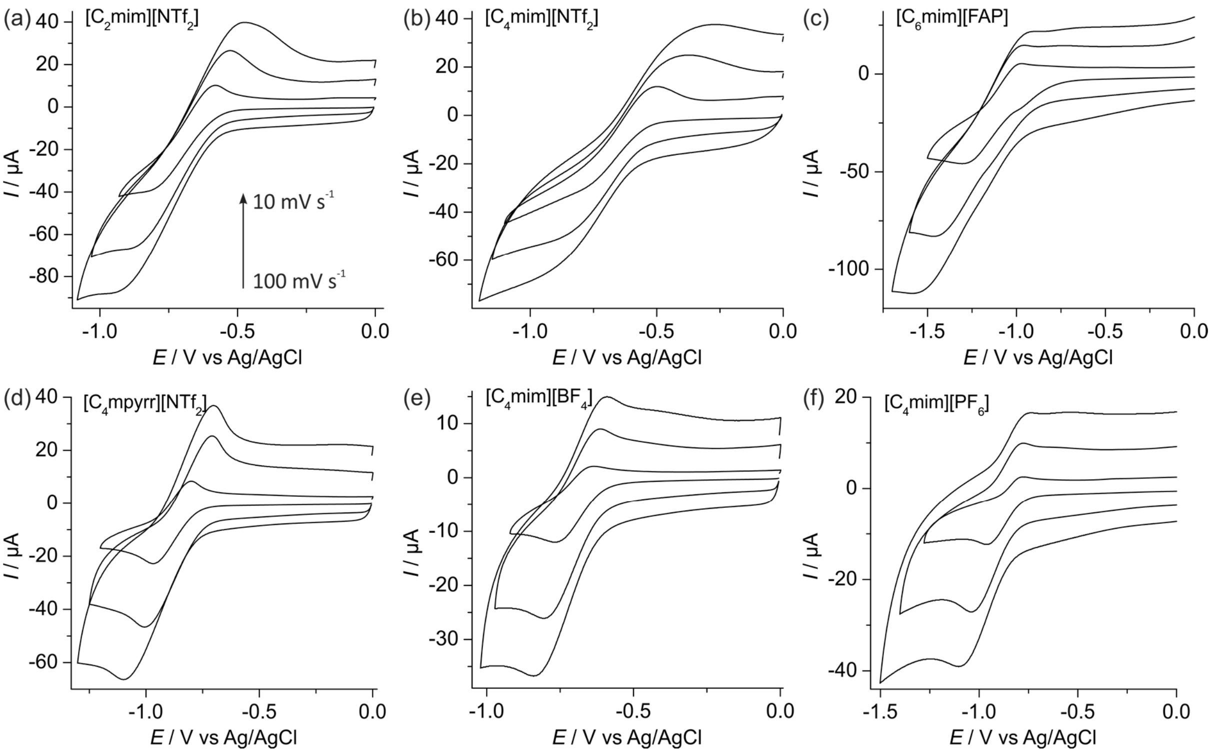

Figure S2. CVs at different scan rates (10, 50, and 100 mV·s^{-1}) for the reduction of 100% vol. oxygen on a SPGE in the six RTILs used in this study.

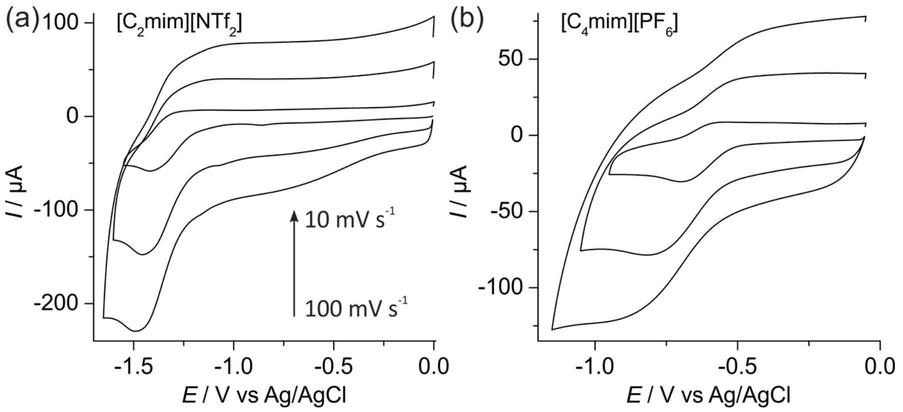

Figure S3. CVs at different scan rates (10, 50, and 100 mV·s^{-1}) on DropSens C-SPEs for 100% vol. O_2 in (**a**) [C$_2$mim][NTf$_2$] and (**b**) [C$_4$mim][PF$_6$].

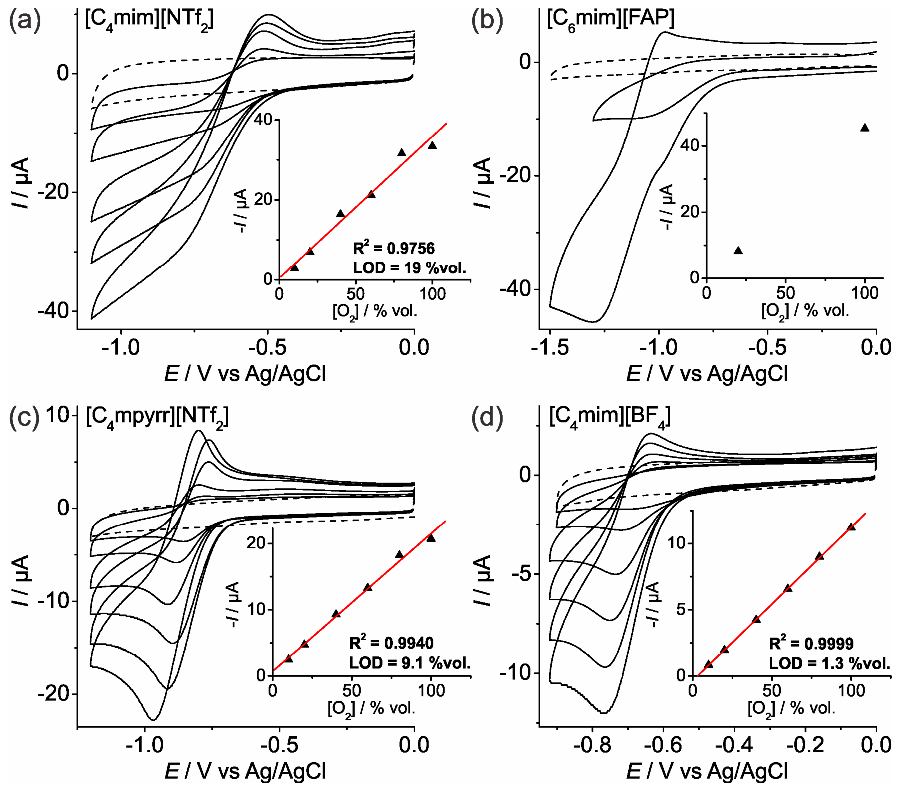

Figure S4. CVs at 10 mV·s^{-1} on a SPGE for different concentrations of oxygen (10–100% vol.) in the four other RTILs (not shown in the main text). The inset shows the respective calibration plots with the R^2 and the limit of detection (LOD) values.

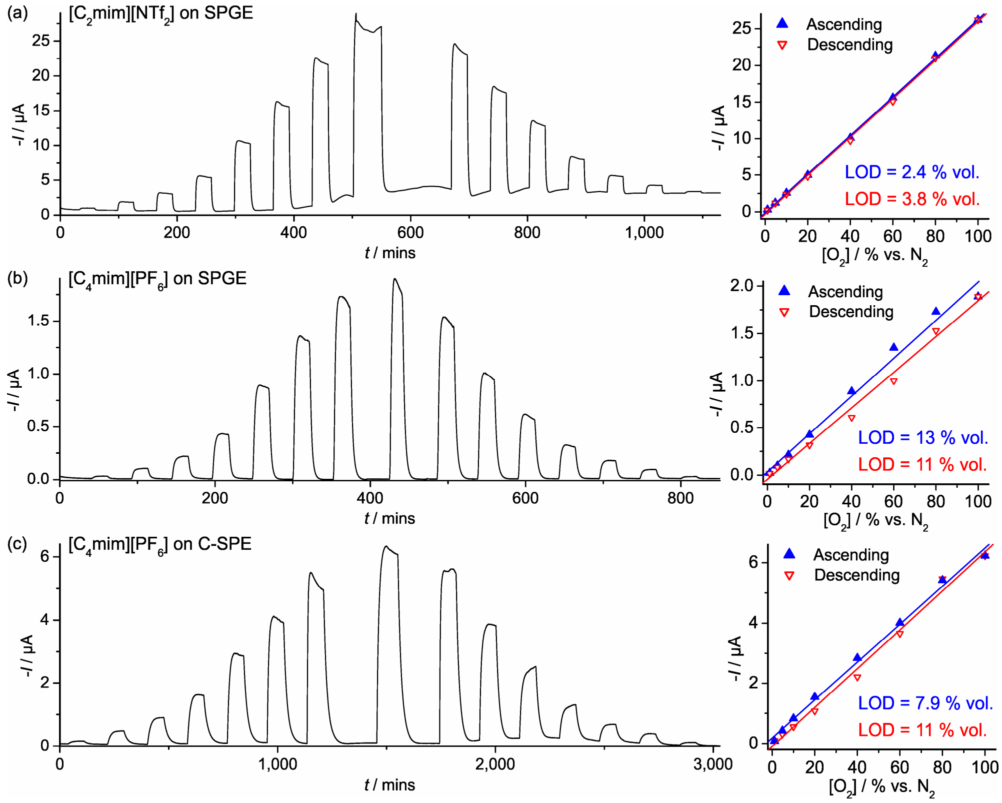

Figure S5. (**Left**) LTCA for different concentrations of oxygen in (**a**) [C2mim][NTf2] on a SPGE, (**b**) [C4mim][PF6] a SPGE, and (**c**) [C4mim][PF6] on a DropSens C-SPE for 1, 5, 10, 20, 40, 60, 80, 100, 80, 60, 40, 20, 10, 5, and 1% vol. O_2 alternating with periods of N_2 purging, performed after running the calibration experiments at the lower (0.1–20% vol. O_2) concentration range on the same system, conducted at 0.15 to 0.20 V negative to the O_2 reduction peak potentials. LTCA for [C2mim][NTf2] on DropSens C-SPE were not conducted as the response had deteriorated during experiments at the lower concentration range. (**Right**) Calibration plots for the initial descending and subsequent ascending change in O_2 concentrations.

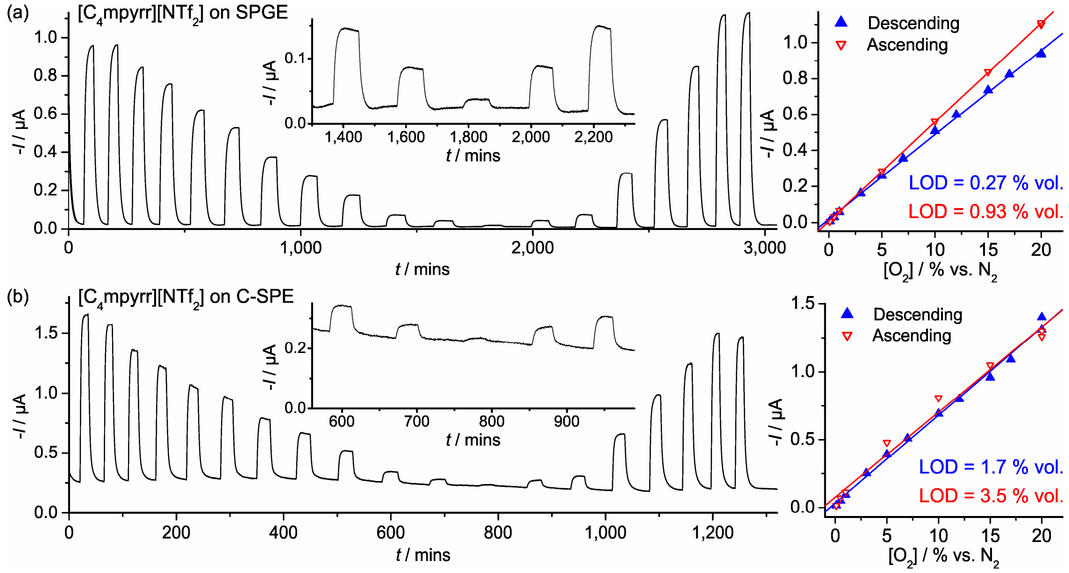

Figure S6. LTCA for different concentrations of oxygen in [C4mpyrr][NTf2] on (**a**) a SPGE and (**b**) a DropSens C-SPE for 20, 20, 17, 15, 12, 10, 7, 5, 3,1,0.5, 0.1, 0.5, 1, 3, 5, 7, 10, 12, 15, 17, 20, and 20% vol. O_2 alternating with periods of N_2 purging, conducted at 0.15 to 0.20 V negative to the O_2 reduction peak potentials. The inset shows a plot zoomed into the lowest concentrations (1, 0.5, 0.1, 0.5, and 1% vol. O_2). The respective calibration plots for the initial descending and subsequent ascending change in O_2 concentrations are shown on the right.

S1 Ammonia detection on SPGEs and C-SPEs

S1.1. Experimental

Ammonia gas (NH_3, 500 ppm, nitrogen fill) was purchased from CAC gases. A step potential of 2.5 mV and a scan rate of 10 mV·s^{-1} was used for cyclic voltammetry (CV) experiments. Chronoamperometric transients on the SPEs employed a sampling time of 0.01 s. For ammonia, CV peak currents were not analysed due to the absence of a clear peak. Instead, currents were extracted from short-term and long-term chronoamperometry experiments to form calibration graphs. In all cases, the blank response (0 ppm ammonia) was subtracted from the analyte response. Ca. 15 minutes was typically sufficient to saturate 20/30 µL of the neat RTIL.

For CV and LTCA experiments, a concentration range between 10–100 ppm for NH_3 was used, with the NH_3 flow rate varying from 20 to 50 sccm, and the N_2 flow rate varying from 980 to 20 sccm. Longer experiments were avoided for NH_3 due to the high cost of the ammonia gas cylinder.

S1.2. Results and Brief Discussion

Oxidation peaks for ammonia were not obvious (or reproducible) on the SPGE surface (see Figures S7 and S8), suggesting that CV is not a suitable technique for ammonia sensing on these electrodes. For ammonia, LTCA responses (Figures S9 and S10) and PSCA responses (Figures S11 and S12) were not ideal in shape, but produced linear calibration graphs with LODs below the permissible exposure limit. Overall, SPGEs with [C_2mim][NTf_2] gave the best analytical responses for NH_3 sensing, but neither electrode material appears to be as suitable for ammonia sensing compared to platinum or gold SPEs (see K. Murugappan, J. Lee and D. S. Silvester, *Electrochem. Commun.*, 2011, **13**, 1435-1438.)

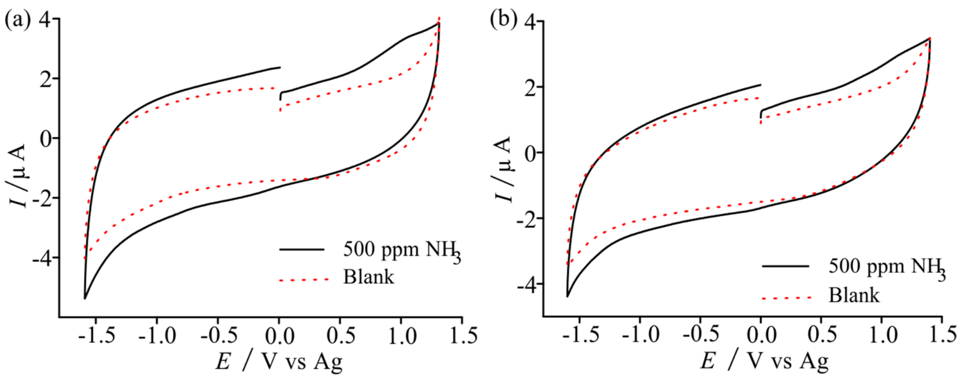

Figure S7. CVs at 10 mV·s^{-1} for 500 ppm NH_3 oxidation in (**a**) [C_2mim][NTf_2] and (**b**) [C_4mim][PF_6] on a SPGE. Blank CVs (in the absence of ammonia) are shown as red dashed lines.

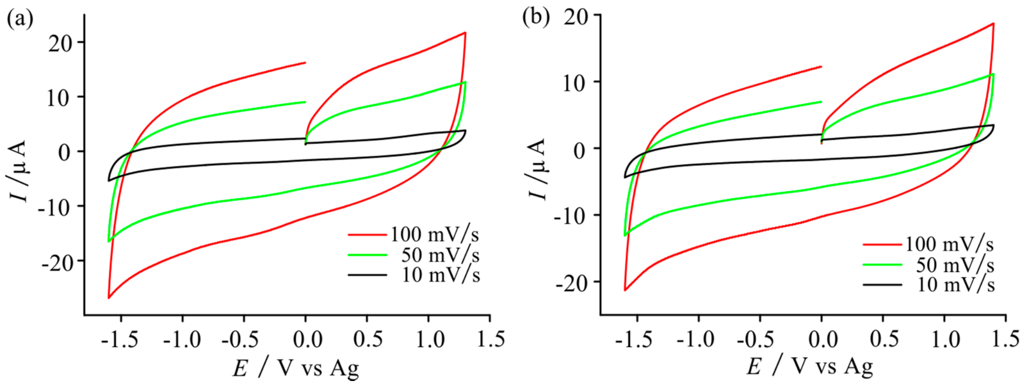

Figure S8. CVs at different scan rates (10, 50, and 100 mV·s^{-1}) for 500 ppm NH_3 in (**a**) [C_2mim][NTf_2] and (**b**) [C_4mim][PF_6] on a SPGE.

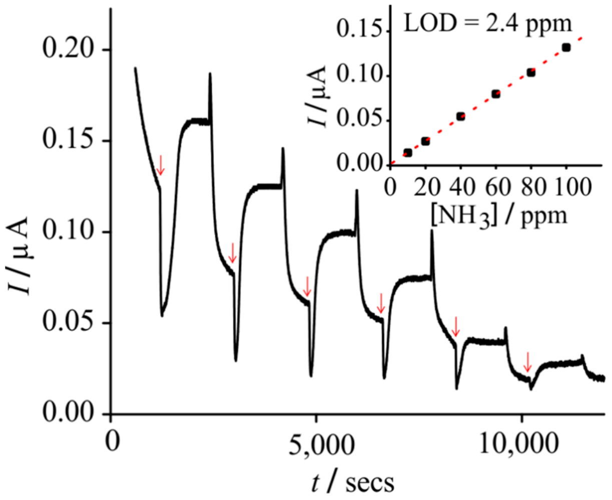

Figure S9. LTCA for 10–100 ppm NH_3, alternating with periods of nitrogen purging, in [C_2mim][NTf_2] RTIL on a SPGE. The potential was biased at +1.25 V. Red arrows indicate the addition of ammonia (100, 80, 60, 40, 20, and 10 ppm) into the cell. The inset shows the corresponding calibration plot and LOD value. Currents were measured from the spike to the steady-state (or maximum) current value.

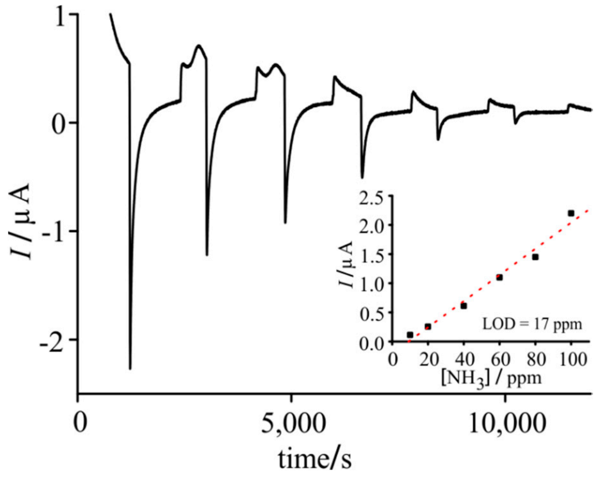

Figure S10. LTCA for 10–100 ppm NH_3 in [C_2mim][NTf_2] on a DropSens C-SPE. The potential was biased at +1.25 V vs. Ag, and the cell was flushed with nitrogen between each NH_3 concentration change.

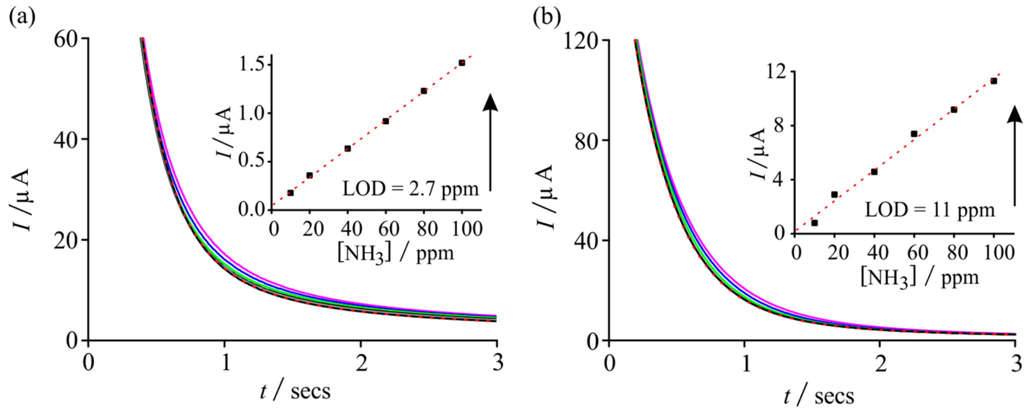

Figure S11. PSCA for 10–100 ppm NH_3 on a SPGE in (**a**) [C_2mim][NTf_2] and (**b**) [C_4mim][PF_6]. The potential was stepped from 0.0 V to +1.25 V and monitored for 10 seconds, although the first three seconds are shown in the figure for clarity. The blank response in the absence of ammonia is shown as the red dotted line. Currents were measured at a fixed time of 2.0 seconds after the potential step. The insets show the (background subtracted) calibration plots and LOD values.

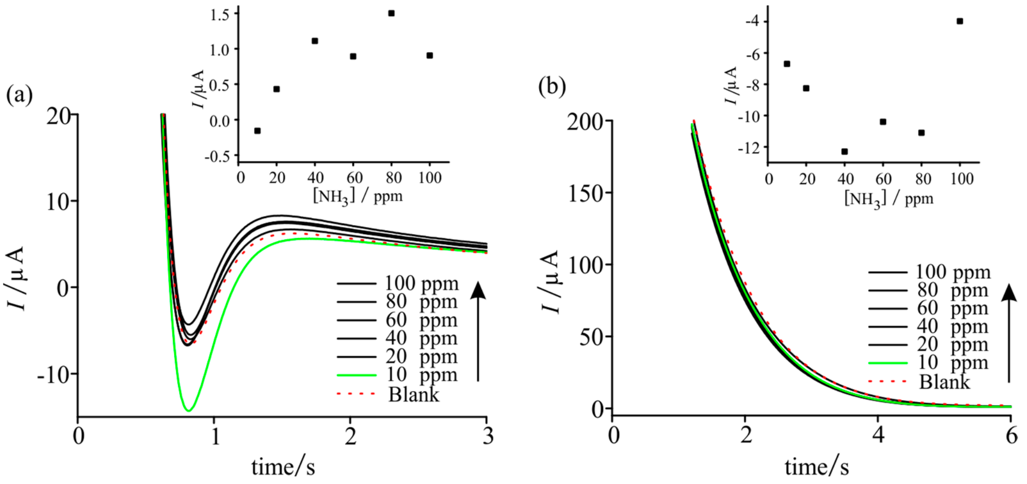

Figure S12. PSCA for 10–100 ppm NH_3 in (**a**) [C_2mim][NTf_2] and (**b**) [C_4mim][PF_6] RTILs on a DropSens C-SPE. The potential was stepped from 0 V to +1.25 V and measured for 10 seconds (only the first three and six seconds, respectively, are shown in the Figure for clarity). Currents for the calibration graphs were measured at 2 s.
